# Observing Pain in Individuals with Cognitive Impairment: A Pilot Comparison Attempt across Countries and across Different Types of Cognitive Impairment

**DOI:** 10.3390/brainsci11111455

**Published:** 2021-11-02

**Authors:** Miriam Kunz, Petra Crutzen-Braaksma, Lydia Giménez-Llort, Sara Invitto, Gaya Villani, Marina deTommaso, Laura Petrini, Lene Vase, Susan Tomczak Matthiesen, Hanne Gottrup, Jone Ansuategui Echeita, Stefan Lautenbacher, Ruth Defrin

**Affiliations:** 1 Department of Psychology and Sociology, Medical Faculty, University of Augsburg, Universitystreet 2, 86135 Augsburg, Germany; 2 Department of General Practice and Elderly Care Medicine, University Medical Center Groningen (UMCG), University of Groningen, 9713 Groningen, The Netherlands; petra_braaksma@live.nl; 3 Department of Psychiatry and Forensic Medicine, School of Medicine, Universitat Autònoma de Barcelona, 08193 Barcelona, Spain; Lidia.Gimenez@uab.cat; 4 Institut de Neurociències, Universitat Autònoma de Barcelona, 08193 Barcelona, Spain; 5 INSPIRE Lab, Department of Biological and Environmental Sciences and Technologies, University of Salento, 73100 Lecce, LE, Italy; sara.invitto@unisalento.it (S.I.); gayavillani@icloud.com (G.V.); 6 Applied Neurophysiology and Pain Unit, Bari Aldo Moro University, 70121 Bari, BA, Italy; marina.detommaso1@gmail.com; 7 Center for Neuroplasticity and Pain (CNAP), Department of Health Science and Technology, Faculty of Medicine, Aalborg University, 9220 Aalborg, Denmark; lap@hst.aau.dk; 8 Division for Psychology and Neuroscience, Department of Psychology and Behavioral Sciences, School of Business and Social Sciences, Aarhus University, 8210 Aarhus, Denmark; lenevase@psy.au.dk (L.V.); stm@psy.au.dk (S.T.M.); 9 Department of Neurology, Demential Clinic, Aarhus University Hospital, 8200 Aarhus, Denmark; hanngott@rm.dk; 10 Department of Rehabilitation Medicine, University Medical Center Groningen, University of Groningen, 9713 Groningen, The Netherlands; j.ansuategui.echeita@umcg.nl; 11 Department of Physiological Psychology, University of Bamberg, 96047 Bamberg, Germany; stefan.lautenbacher@uni-bamberg.de; 12 Department of Physical Therapy, School of Health Professions, Sackler Faculty of Medicine, Tel Aviv University, Tel Aviv 6997801, Israel; rutidef@post.tau.ac.il

**Keywords:** pain, cognitive impairment, facial expression, cultural differences, dementia, mild cognitive impairment, intellectual disability, pain observation, PAIC

## Abstract

Facial expression is a key aspect in observational scales developed to improve pain assessment in individuals with cognitive impairments. Although these scales are used internationally in individuals with different types of cognitive impairments, it is not known whether observing facial expressions of pain might differ between regions or between different types of cognitive impairments. In a pilot study, facial responses to standardized experimental pressure pain were assessed among individuals with different types of cognitive impairments (dementia, mild cognitive impairment, Huntington’s disease, and intellectual disability) from different countries (Denmark, Germany, Italy, Israel, and Spain) and were analyzed using facial descriptors from the PAIC scale (Pain Assessment in Impaired Cognition). We found high inter-rater reliability between observers from different countries. Moreover, facial responses to pain did not differ between individuals with dementia from different countries (Denmark, Germany, and Spain). However, the type of cognitive impairment had a significant impact; with individuals with intellectual disability (all being from Israel) showing the strongest facial responses. Our pilot data suggest that the country of origin does not strongly affect how pain is facially expressed or how facial responses are being scored. However, the type of cognitive impairment showed a clear effect in our pilot study, with elevated facial responses in individuals with intellectual disability.

## 1. Introduction

Assessing pain in individuals with cognitive impairments can be quite challenging, given that the ability to provide a valid self-report of pain can be limited and deteriorates across the course of cognitive decline [1,2,3]. As a result, pain is often overlooked in individuals with cognitive impairments and, thus, remains undertreated [4]. To improve this situation, observational pain scales that rely on non-verbal indicators, such as facial expression, to assess pain have been developed [2,5,6]. One of these scales is the PAIC scale (“Pain Assessment in Individuals with Cognitive impairment”), which was recently developed under international consensus out of an EU-COST collaboration between researchers from different disciplines (e.g., medicine, nursing, physiotherapy, psychology, and neuroscience) and from different European regions with different languages, ethnicity, and culture [7,8,9]. The aim was to develop an internationally agreed-upon tool to assess pain, which is culturally and linguistically robust, for individuals with cognitive impairment by selecting items out of existing observational scales and critically re-assessing their suitability to detect pain. Within the category of facial responses (facial responses are the most prominent non-verbal pain behaviors), 15 facial descriptors were identified that had been frequently used to assess pain states in individuals with cognitive impairments [9,10].

When intending to use observational pain scales—such as the PAIC—to assess pain reliably and validly in individuals with cognitive impairments, the question arises whether this is possible regardless of the region where the assessment takes place or regardless of the type of cognitive impairment of the individual being assessed. It is possible that cultural aspects might influence not only how pain is facially expressed but also how these facial expressions are judged by observers [11,12]. Within the communications model of pain [13,14], these two aspects, namely the facial expression of pain on the one hand and the judgment of the facial expressions by observers on the other hand, are conceptualized as the encoding and decoding of pain, respectively. As culture, ethnic, and linguistic aspects may shape the individual’s responses and believes towards pain [15,16], these aspects could influence the facial encoding as well as the decoding process of pain. Moreover, facial responses to pain (encoding) might also differ between individuals with different types of cognitive impairments. 

How culture and type of cognitive impairment affect the facial encoding and decoding of pain have not been systematically investigated so far. With the present study, we aim to do so by (i) investigating whether observational pain assessment might differ between various European countries, not all sharing the same culture, ethnicity, and language [17,18], and (ii) between different types of cognitive impairments. To this aim, a standardized experimental pain protocol was developed and applied in different European countries (north, central, and south) and in one western Asian country (Israel); and in individuals with different types of cognitive impairments (neurodegenerative and developmental disorders). Facial responses of individuals from these different countries and these different types of cognitive impairments to the experimental pain (encoding) were videotaped, and observers from different countries assessed the pain responses using the facial descriptors from the PAIC scale (decoding). Given that this is only a pilot comparison study, country differences in facial encoding were only realized between the countries Denmark, Germany, and Spain, given that only in these three countries a common cognitive impairment (dementia) was investigated. With regard to the comparison between types of cognitive impairments, individuals with certain types of cognitive impairments sometimes only stemmed from one country in this pilot attempt; thus, the type-of-cognitive-impairment comparison is confounded by country in our study.

## 2. Materials and Methods

A standardized experimental pain protocol (pressure pain) was carried out in five different research sites in four European countries (i.e., Denmark, Germany, Italy, and Spain) and in one western Asian country (Israel), including individuals with varying types and degrees of cognitive impairments (i.e., neurodegenerative disorders: mild cognitive impairment, dementia, and Huntington’s disease; developmental disorder: intellectual disability; and cognitively healthy controls). During pressure stimulation, the faces of the participants were videotaped, and afterward, observers rated the facial responses displayed by the participants using the facial descriptors of the PAIC scale [9].

### 2.1. Participants

In total, 102 individuals with varying types of cognitive impairments and 82 cognitively healthy controls participated in the study (see Table 1). Recruitment was carried out in five countries, namely in Denmark, Germany, Israel, Italy, and Spain. In Denmark, Germany, and Spain, older individuals with mild cognitive impairments (MCI) or dementia, and age-matched cognitively healthy individuals were recruited (given the participating centers’ health care expertise in the field of pain and dementia). In Israel, individuals with intellectual disabilities were recruited (given the participating health care center’s expertise in the field of pain and intellectual disability). In Italy, individuals with Huntington’s disease were recruited (given the participating health care center’s expertise in the field of pain and Huntington’s disease). We excluded individuals with Parkinson’s disease, schizophrenia, and stroke patients with facial impairments, as these conditions could impair facial responses. Moreover, the degree of cognitive impairment was mostly only mild to moderate so that verbal report could be assessed in the majority of participants (see % missing ratings in Table 1). Demographic information on all participants is displayed in Table 1. Ethics approval was obtained for each of the five centers separately, consistent with local procedures, at the local ethics committee. Written informed consent from the participants and/or (when appropriate) from a legal guardian (e.g., family) was obtained for all participants. 

Experimental pain testing: For experimental pain induction, pressure stimuli of varying intensities were used that were applied by a hand-held pressure algometer with a probe area of 1 cm^2^ [Somedic (Hörby, Sweden) or Fisher (Wagner Pain Test, Greenwich, CT, USA)]. Following established protocols, three different pressure intensities were used (50, 200, and 400 kPa) that have been shown to elicit “no pain”, “slight”, and “moderate pain” sensations, respectively [19,20,21,22]. Pressure stimuli were applied to the midpoint of the upper border of the trapezius muscle (back shoulder area) in ascending order. An ascending order was chosen for three reasons: (1) to reduce anxiety in participants, (2) avoid carry-over effects, and (3) to be able to immediately stop the stimulation protocol if the stimulation was too painful (this did not occur in the present sample). Stimuli were applied to the right and left shoulder, thus resulting in 2 (left and right) × 3 (intensities) = 6 pressure stimuli. The pressure was increased steadily for 2 s until the desired intensity was reached and was then kept constant for another 5 s. To ensure that the experimental pain testing was conducted similarly across sites, the stimulation protocol was trained in a combined session where representatives from all sites participated, who later trained additional experimenters if necessary. Moreover, a written testing protocol was handed out to all experimenters detailing the experimental procedure. No more than two experimenters per site conducted the testing.

The faces of the participants were videotaped during the whole stimulation, allowing a frontal view of the face. The start of each pressure stimulation was verbally indicated. Immediately after each stimulus, participants were asked to rate the pain sensation. Per site, different rating scales were used, depending on the type of patients being investigated or depending on the standard protocol of that site, respectively. In Germany and Italy, a five-category verbal rating scale (no pain, mild pain, moderate pain, strong pain, and very strong pain) was used. In Denmark and Spain, a numerical rating scale (0–10) was used, and in Israel, a Pyramid scale (0–5) was used. If participants could not provide a rating, the investigator recorded this event and applied the next stimulus. 

### 2.2. Observers

Each research site where the experimental pain testing was conducted, recruited at least one observer who rated the videos recorded in that research site. Table 1 gives an overview of the observers’ characteristic separately for each research site/country. 

There were two observers (Germany) who did not only rate the videos of their own research site, but rated all videos included in this study (videos of all the 184 participants). They served as a kind of a “gold standard”, because the German research site has had a long-standing expertise in facial pain decoding, and therefore, has become the reference center in the present study. Thus, all observers’ ratings from other countries could be compared with the German ratings to assess inter-rater-reliability across countries. The German raters were blind to observer ratings from the other countries.

Rating of the video recording: Observers were asked to rate the videos using 15 facial descriptors of pain (part of the research version of the PAIC scale). As described in detail before [9,10], these 15 facial descriptors were selected from established observational pain assessment tools for people with dementia. These 15 facial descriptors are listed in Table 2. Facial descriptors were scored on a four-point category scale, describing how well the descriptor item coincided with the facial response of the videotaped individual (not at all, slight degree, moderate degree, and great degree). The PAIC tool has been translated into eight languages (www.PAIC15.com, accessed date: 1 November 2021), so that the scoring was mostly carried out in the native language of the country (German, Italian, and Spanish), with the exception of Israel and Denmark, where the original English version of the PAIC scale was used (a validated Danish version has become available in the meantime).

The observers watched the video of a participant, and after each pressure stimulus, the video was paused to allow the observers to provide their ratings using the 15 facial descriptors of pain (each video was only watched once). Altogether, the observer rated facial responses to six pressure stimuli per observed person. Ratings were carried out using paper sheets of the PAIC.

### 2.3. Statistics

The statistical analyses were carried out in three steps.

Step 1: Pre-analyses—item selection: In order to narrow down the number of facial descriptors for further analyses (comparisons between countries and between types of cognitive impairments) to those items that are best suitable to assess pain in the present context, we excluded items based on floor effects and poor reliability. 

The floor effect was defined as facial descriptors scored in less than 15% of the responses to the strongest pressure stimuli, namely 400 kPa. Given that this pressure intensity is known to elicit painful sensations in most individuals, items that are rarely observed during this noxious stimulus, do not seem suitable to assess pain in our context. We defined an item as being observed if it was scored with a number >0. All observer ratings (from all countries) were used for this analysis.

Poor reliability of an item was based on the agreement (ICC; two-way random-effects model) of the two observers from Germany who rated all videos. For the reliability analyses, we again focused on the responses to the 400 kPa pressure stimuli, given that this was the highest pain intensity that elicited most facial responses, and thus, provided enough variance to conduct ICC analyses. Poor reliability was defined as facial descriptors with an intraclass correlation coefficient (ICC) less than 0.4 [23]. 

Only if an item survived both exclusion criteria (“floor effect” and “poor ICC”), it was included in the next step.

Step 2: Differences between observers (decoding of pain): To investigate whether the scoring of the facial descriptors differs between observers from different countries, we (i) conducted inter-rater reliability between the German observers (given that these rated all videos, and served as “gold standard”) and observers from the other countries (who only rated the videos recorded in their own country) and (ii) conducted t-tests to compare the magnitude of the scores between “gold standard” observers and observers from the other countries. We again focused on the responses to the 400 kPa pressure stimuli (sum score of the selected facial descriptors from step 1), given that this was the highest pain intensity that elicited most facial responses. Per country, scores were averaged across observers from that country. 

Step 3: Differences between observed participants (encoding of pain): To investigate whether pain responses differ between observed participants from (a) different countries (in case of individuals with dementia) and between (b) different types of cognitive impairment, analyses of variance with repeated measurements (50, 200, 400 kPa) were conducted with the between-subject factor (a) “country” and (b) “type of cognitive impairment”, respectively. As dependent variables, the sum score of the selected facial descriptors (see step 1) was used and scores of different observers (both within and across countries) were averaged. In case of significant findings, the single facial descriptors were compared between “country” and “type of cognitive impairment, respectively, in post-hoc analyses to see whether differences were due to certain types of facial responses. 

Analyses were conducted with SPSS 26, and the alpha was set to 0.05.

## 3. Results

### 3.1. Step 1: Pre-Analyses—Item Selection

Table 2 shows the frequency scores of the 15 facial descriptors based on all observer ratings of facial responses to 400 kPa. As can be seen, seven items were only rarely observed (<15%), and thus, they are not indicative for pain in the present context and were, therefore, excluded from further analyses. The ICC scores for the remaining facial descriptors (based on the German observers who rated all videos) are also listed in Table 2. For six of the remaining facial descriptors, at least moderate reliability scores were found (ICC > 0.4), and these items (items 1, 2, 3, 4, 6, 13) were retained for further analyses (see step 2 and step 3). For these analyses, the scores of the remaining facial descriptors were summed up to form a SUM-score of facial responses. This selection outcome, namely retaining six out of 15 items, is very comparable to previous attempts [7].

### 3.2. Step 2: Differences between Observers (Decoding of Pain)

**Figure 1:**
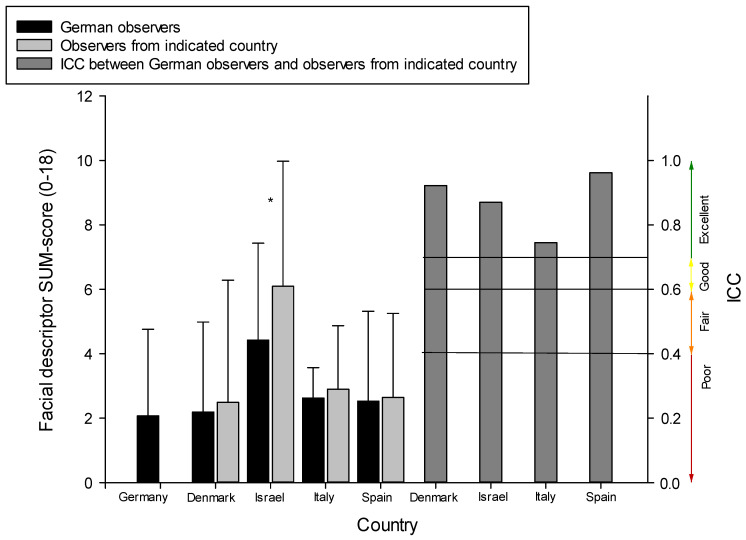
Step 2; decoding–comparison between countries: Mean SUM-scores (SD) of observer ratings for facial responses to 400 kPa pressure (highest pain intensity). Scores are given separately for the German observers (who scored all videos; “gold-standard”) and the observers from each given country. ICC scores (reliability) are presented at the right side between German observers and observers from each given country. The symbol * indicates *p* < 0.05.

Given that only the German observers rated all videos, we always compared the German observer ratings (“gold standard”) with the observer ratings from the other countries. For this step, we focused on the SUM-scores of facial responses to the highest pressure intensity (400 kPa). The reliability scores (ICC) between observers from different countries were excellent, as can be seen in Figure 1 (right side), with minor variations between countries. Moreover, observers from different countries also reached comparable SUM-scores when judging the facial responses (see the left side of Figure 1). Scores of the German observers did not differ significantly from the scores of observers from the other countries (see Figure 1), with only one exception. This exception occurred between German and Israel observers, with Israel observers rating the facial responses slightly higher than the German observers (T(68) = −2.03; *p* = 0.047). 

It is also remarkable in Figure 1 that the SUM-scores for the ID participants from Israel are much higher than those of all other groups, a finding which will be addressed in the next section.

### 3.3. Step 3: Differences between Participants (Encoding of Pain)

**Figure 2:**
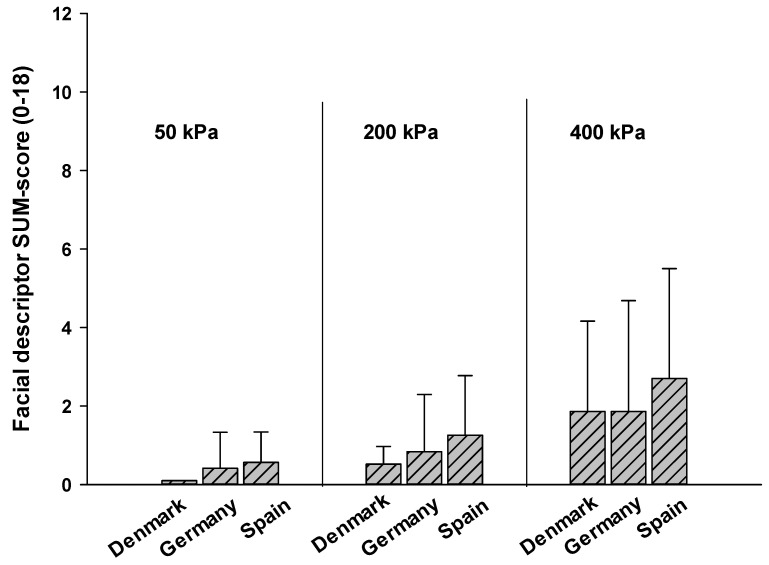
Step 3, encoding—comparison between countries: Comparing mean SUM-scores (SD) of individuals with dementia from different countries.

**Figure 3:**
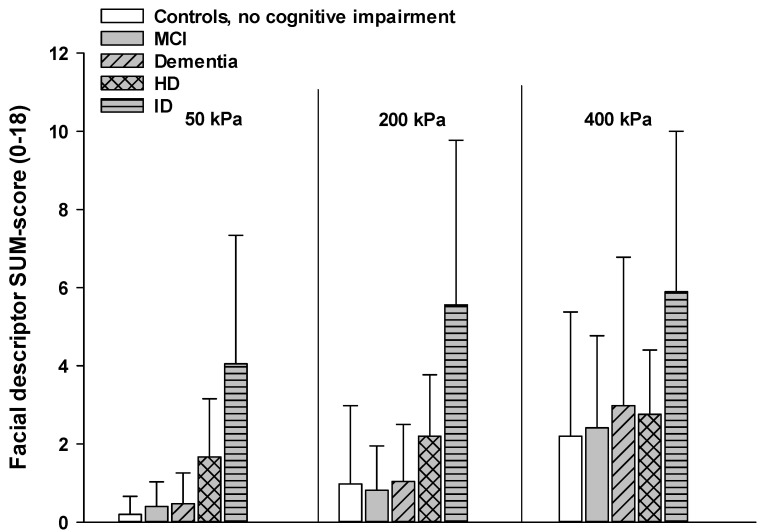
Step 3, encoding—comparison between types of cognitive impairment: Comparing mean SUM-scores (SD) of individuals with different types of cognitive impairments.

a. Comparing individuals only with the diagnosis “dementia” between countries (Denmark, Germany, and Spain): As can be seen in Figure 2, there was a main effect for intensity, with facial responses increasing across pressure intensities (F(2, 88) = 32.41, *p* < 0.001). This increase did not differ between individuals with dementia from different countries (no interaction effect of intensity*country (F(4, 88) = 1.87, *p* = 0.118)), nor was there an overall effect for country (F(2, 44) = 1.35, *p* = 0.264). Thus, individuals with dementia showed similar facial responses to experimentally induced pressure pain across the north, central, and south of Europe.

b. Comparing individuals with different types of cognitive impairments (controls, dementia, ID, MCI, and HD): As can be seen in Figure 3, there was a main effect for intensity, with facial responses increasing across pressure intensities (F (2, 646) = 21.64, *p* < 0.001). This increase did not differ between groups as indicated by a non-significant interaction effect of intensity*cognitive impairment (F (8, 646) = 0.45, *p* = 0.888). However, there was a main effect for group (F (4, 323) = 42.461, *p* < 0.001). Post-hoc analyses revealed that the sum scores of individuals with intellectual disability (ID) at all pressure intensities were higher from the sum scores of all other patient groups (all *p*-values < 0.05).

**Figure 4:**
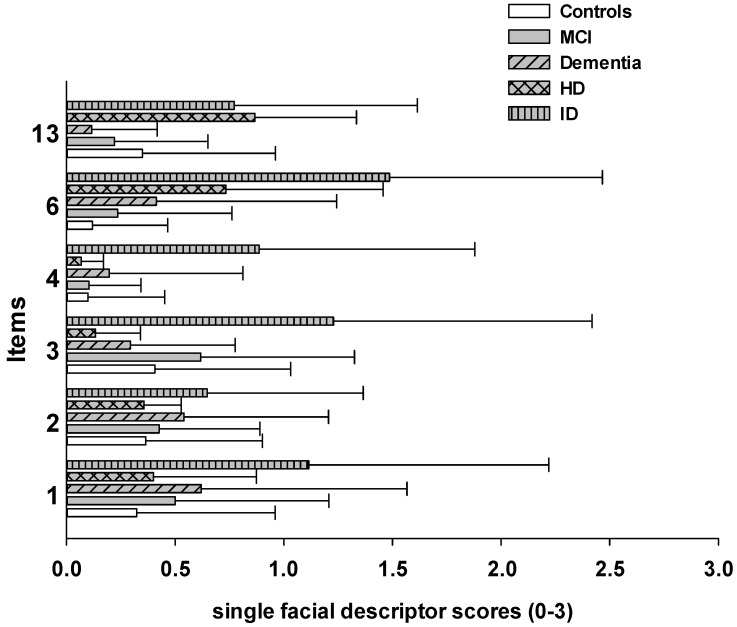
Step 3, encoding—post-hoc comparisons between types of cognitive impairment: Comparing single facial descriptor scores (mean, SD) (during 400 kPa pressure pain) of individuals with different types of cognitive impairments.

To investigate whether this increase in facial responses in the ID group was driven by a specific type of facial response, we compared each single facial descriptor (response to 400 kPa) between groups. As can be seen in Figure 4, individuals with ID responded to painful pressure stimulation, especially with increased narrowing and closing of the eyes (items 3 and 4) as well as with the opening of the mouth (item 6) in comparison to the other groups.

## 4. Discussion

Facial expression is a key aspect in observational scales developed to improve pain assessment in individuals with cognitive impairment. Although these scales are used worldwide across different regions and across different types of cognitive impairments [2], little is known about how these factors might impact observational pain assessment. In this pilot study, we compared observational pain assessment between individuals (experiencing experimental pain) from different European countries and one western Asian country as well as with different types of cognitive impairments. Overall, differences between countries were negligible; this was the case both for the individuals facially expressing pain (encoding; this was only investigated for individuals with dementia) as well as for the observers judging the facial expressions of pain (decoding). Only the type of cognitive impairment had a substantial impact on the facial encoding of pain, with individuals with intellectual impairments (ID) (all being from Israel) showing stronger facial responses to experimental pain. We discuss these pilot findings in detail below.

### 4.1. Facial Encoding and Decoding of Pain: Differences between Countries

Cross-cultural differences are evident in many aspects of human behavior. Specifically with regard to pain, culture has been found to interact with sensitivity to noxious stimuli (e.g., [15]) and with attitudes and expectations towards chronic pain, its expressions and possible remedies [24]. However, it is yet unknown whether culture and associated factors such as language also affect the facial encoding of pain. Indeed, most studies on facial responses to pain have been conducted in culturally, ethnically, and linguistically homogenous samples from northern Europe and in North America, and studies are lacking that have compared facial responses to pain between individuals from more heterogeneous backgrounds [25]. The present comparative study is a first pilot attempt to investigate whether facial encoding and especially decoding of pain differs between patients and observers from a Nordic (Denmark), a western central (Germany), and two European Mediterranean countries (Italy and Spain) as well as from one western Asian country (Israel).

Our pilot data suggest that cross-regional differences might be negligible for the decoding process of rating facial responses to pain. This was indicated by high inter-rater reliability scores between observers from Germany (who scored all videos using the PAIC facial descriptors and who served as kind of a “gold standard”, given the expertise of the center in facial expression decoding) and the observers from the other countries. Besides looking at classical inter-rater reliability, we also compared the overall observational scores between German observers and the observers from the other countries using T-tests in order to investigate whether the magnitude of the scores might differ between countries. We found that the German observers scored the magnitudes of facial responses very similarly to the observers from the other countries. Thus, even the magnitude of scoring the various facial descriptors was very stable despite including very different regions across Europe. The only significant difference we found was between the German observers and the observers from Israel. Observers from Israel reached slightly higher observational scores compared to the German observers. When computing the effect size for this group difference, only a small effect occurred (Cohens’ d = 0.25). Thus, even this cross-regional difference seems negligible.

Regarding regional differences in the facial encoding process of pain, we compared facial descriptor sum scores of individuals with dementia from three different countries (north, central and south of Europe). Only individuals suffering from dementia could be compared between countries because the other types of cognitive impairment (ID and HD) were only assessed within one country. We found very comparable facial responses across experimental pressure intensities in individuals with dementia that did not differ significantly between the three countries. Thus, our pilot data suggest that facial encoding of pain in individuals with dementia does not seem to be markedly influenced by regional differences between different European countries. This finding might seem surprising, given that cultural differences have been shown to play a role in facial expressions [26,27,28], which are due to culture-specific display rules, which regulate how we express emotions and pain. However, recent findings have suggested that the influence of learned social display declines in individuals with dementia [29]. Along with the neurodegenerative decline of frontal structures, the socially learned regulation of facial responses to pain seems to diminish in dementia [22] and, as a consequence, individuals with dementia seem to facially express pain more “unfiltered” compared to cognitively healthy controls. Thus, it is possible that cross-cultural differences in facial encoding of pain are of less relevance—as indicated by our data—in individuals with dementia, who increasingly lose their social regulation of behavior.

### 4.2. Facial Encoding of Pain: Differences between Different Types of Cognitive Impairments

Most observational pain assessment scales have been developed for older individuals with dementia [2]. This is also true for the PAIC scale, with the psychometrical testing being primarily conducted in large samples of individuals with dementia [7,8]. However, cognitive impairments hampering the self-report of pain and, therefore, requiring observational pain assessment scales also occur in other types of cognitive impairment [30]. Nevertheless, direct comparisons of non-verbal pain behaviors between individuals with different types of cognitive impairments is missing so far. Given that we used the same standardized experimental pain protocol, we were able to directly compare the elicited facial responses between different types and different degrees of cognitive impairments (MCI, dementia, ID, HD, and cognitively healthy controls). We found that in all groups, pain-indicative facial responses increased across pressure pain intensities. Thus, the facial descriptors of the PAIC scale seem to validly mirror different pain intensities, regardless of the type or degree of cognitive impairment. Moreover, most groups showed comparable degrees of facial responses, with one exception: individuals with ID (all being from Israel) were facially significantly more responsive than all other groups. To investigate whether this increase was due to an overall increase in facial responses or rather to an increase in certain facial descriptors, we also compared single facial descriptors between groups. Interestingly, especially the more anatomical descriptors, such as “narrowing the eyes”, “closing of the eyes”, and “opening of the mouth”, were markedly increased in this group. Interestingly, the aforementioned facial descriptors have also proven to be indicative of clinical pain [31]. Thus, it is not unspecific grimacing or simply a subjective expression of pain that led to increased scores but an increase in pain-specific facial muscle movements. One might question whether this increase in facial responses to pain in individuals with ID might really be due to the type of cognitive impairment and not simply be an age effect, given that this group was markedly younger than the rest of the sample. However, previous studies could show that the strength of facial responses to pain does not change with age [32]. Moreover, Defrin and colleagues recently showed that pain responses in individuals with ID are much elevated when compared to cognitively healthy age-matched controls [33] (this study was also conducted in Israel). Thus, it is very unlikely that the found group difference in the present study can simply be explained by age differences or by the county of origin. However, what might be the reason for the elevated facial responses in individuals with ID? We can only speculate at this point. Referring to the above mentioned socially learned display rules, it is thinkable that never having learned these display rules renders individuals with ID to be facially much more expressive and unfiltered compared to older individuals with cognitive impairments, who have learned to down-regulate their facial expressions in the past (even though this learned regulation might decline).

### 4.3. Limitations

We have to acknowledge that this is only a pilot study, with sample sizes often being very small, which limits the generalizability of our data. Although we tried to compare observational pain assessment between patients and observers from geographically different European regions, we cannot claim that we have captured and analyzed all the possible heterogeneity that is present in this part of the world. Our approach was a first pilot attempt and should be broadened to different regions in the world. Moreover, we did not systematically assess the ethnic and cultural background of the participants, and thus, we cannot exclude that some participants in each country differed in their cultural background. Furthermore, even though we used a standardized pain protocol developed and practiced at a combined meeting, there was some heterogeneity between studies with regard to the type of rating scales used (e.g., Israel being the only country using a Pyramid rating scale) and with regard to the language in which the PAIC was used (some countries used the PAIC in their native language, whereas others used the original English version). In our pilot approach, we could not recruit equal numbers of different diagnoses over different countries, which does not allow us to directly compare country versus diagnosis effects. Given that certain diagnoses were only assessed in one country, the comparison between types of diagnosis is unfortunately confounded by the country of origin of the participants.

## 5. Conclusions

Our pilot study suggests that facial encoding and decoding of pain in individuals with cognitive impairments seems very stable across various European regions and one western Asian country, which seems to support the notion that pain speaks one common facial language. This also supports up-to-date clinical research approaches to develop observational pain scales for individuals with cognitive impairment that can be used worldwide for different regions by supplying the necessary validated translations. For use of observational scales in clinical practice, it is important to apply these scales in transfer or during movement situations, given that here the probability of painful sensations that elicit increased facial responses [34].

## Figures and Tables

**Table 1:** Demographic data of the observers as well as of the participants.

	Countries								
	Denmark		Germany			Israel	Italy	Spain	
**Observers**									
N	1		2 *			2	2	2	
Mean Age	23.0		23.5			25.5	23.0	37.5	
Sex (female/male)	1/0		1/1			2/0	2/0	2/0	
**Participants**									
Diagnosis	controls	Dementia	controls	MCI	Dementia	ID	HD	Controls	Dementia
N	19	6	44	18	18	27	13	19	20
Mean Age (SD)	73.6 (5.7)	67.2 (9.6)	71.1 (3.9)	73.3 (5.3)	74.1 (5.1)	35.3 (6.2)	58.8 (10.4.)	65.0 (4.5)	78.0 (6.8)
Sex (female/male)	13/6	4/2	23/21	10/8	11/7	13/14	7/6	16/3	13/7
Self-report ratings (mean (SD)) **									
50	0.6 (0.7)	0.8 (1.5)	1.1 (0.2)	1.2 (0.3)	1.1 (0.3)	1.4 (1.6)	0.0 (0.0)	0.6 (0.6)	1.4 (2.7)
200	4.5 (2.4)	3.1 (2.3)	1.9 (0.8)	1.9 (0.7)	2.2 (0.8)	2.6 (1.7)	0.0 (0.0)	2.8 (1.8)	2.1 (3.2)
400	6.3 (2.7)	6.2 (3.1)	3.1 (0.7)	3.3 (0.8)	3.4 (0.9)	3.0 (1.5)	0.0 (0.0)	5.1 (2.6)	3.9 (3.9)
% missing ratings	0%	0%	0%	0%	10%	7%	0%	0%	6%

Abbreviations: MCI = mild cognitive impairment; IDD = Intellectual and developmental disability; HD = Huntington’s disease. * Both German observers scored all videos from all countries, the other observers only scored the videos from their own country. ** different rating scales were used across countries: Denmark and Spain = Numerical rating scale (0–10); Germany and Italy = verbal category scale (0–5); Israel = Pyramid scale (0–5).

**Table 2:** Step 1 (item selection): Facial descriptor frequency and reliability.

Facial Descriptor		Frequency (%) *	ICC **
1	Pained expression	**31**	**0.74**
2	Frowning	**34**	**0.58**
3	Narrowing eyes	**31**	**0.70**
4	Closing eyes	**20**	**0.60**
5	Raising upper lip	**27**	0.29
6	Opened mouth	**33**	**0.78**
7	Thightened lips	**22**	0.33
8	Clenched teeth	10	-
9	Empty gaze	11	-
10	Seeming disinterested	6	-
11	Pale face	5	-
12	Teary eyed	6	-
13	Looking tense	**21**	**0.45**
14	Looking sad	7	-
15	Looking frightened	8	-

* Frequency of scoring the facial descriptor across all types of cognitive impairment and all countries, (decoding of 400 kPa pressure pain responses); ** ICC of the scores of German observer 1 and German observer 2 (decoding of 400 kPa pressure pain responses).

## Data Availability

The descriptive data of the study (observer ratings) can be provided by contacting the corresponding author of the study.

## References

[1] Achterberg W., Lautenbacher S. (2017). Editorial: Pain in Dementia: A Distressing Combination of Several Factors. Curr. Alzheimer Res..

[2] Herr K., Coyne P.J., Ely E., Gélinas C., Manworren R.C. (2019). Pain Assessment in the Patient Unable to Self-Report: Clinical Practice Recommendations in Support of the ASPMN 2019 Position Statement. Pain Manag. Nurs..

[3] Lautenbacher S., Kunz M. (2019). Assessing pain in patients with dementia. Schmerz.

[4] Achterberg W.P., Erdal A., Husebo B.S., Kunz M., Lautenbacher S. (2021). Are Chronic Pain Patients with Dementia Being Undermedicated?. J. Pain Res..

[5] Kappesser J., Voit S., Lautenbacher S., Hermann C. (2020). Pain assessment for cognitively impaired older adults: Do items of available observer tools reflect pain-specific responses?. Eur. J. Pain.

[6] Schofield P., Abdulla A. (2018). Pain assessment in the older population: What the literature says. Age Ageing.

[7] Kunz M., de Waal M.W., Achterberg W.P., Gimenez-Llort L., Lobbezoo F., Sampson E.L., Defrin R., Invitto S., Konstantinovic L., Lautenbacher S. (2020). The Pain Assessment in Impaired Cognition Scale (PAIC15): A multidisciplinary and international approach to develop and test a meta-tool for pain assessment in impaired cognition, especially dementia. Eur. J. Pain.

[8] de Waal M.W., van Dalen-Kok A.H., de Vet H.C., Gimenez-Llort L., Konstantinovic L., de Tommaso M., Fischer T., Lukas A., Kunz M., Achterberg W.P. (2020). Observational pain assessment in older persons with dementia in four countries: Observer agreement of items and factor structure of the pain assessment in impaired cognition. Eur. J. Pain.

[9] Corbett A., Achterberg W.P., Husebo B.S., Lobbezoo F., De Vet H., Kunz M., Strand L.I., Constantinou M., Tudose C., Kappesser J. (2014). An international road map to improve pain assessment in people with impaired cognition: The development of the Pain Assessment in Impaired Cognition (PAIC) meta-tool. BMC Neurol..

[10] Lautenbacher S., Walz A.L., Kunz M. (2018). Using observational facial descriptors to infer pain in persons with and without dementia. BMC Geriatr..

[11] Turk D.C., Okifuji A. (1999). Assessment of patients’ reporting of pain: An integrated perspective. Lancet.

[12] Peacock S., Patel S. (2008). Cultural Influences on Pain. Rev. Pain.

[13] Hadjistavropoulos T., Craig K. (2002). A theoretical framework for understanding self-report and observational measures of pain: A communications model. Behav. Res. Ther..

[14] Rosenthal R., Scherer K., Ekman P. (1982). Conducting Judgment Studies. Handbook of Methods in Nonverbal Behavior Research.

[15] Rahim-Williams B., Riley J.L., Williams A.K., Fillingim R.B. (2012). A quantitative review of ethnic group differences in experimental pain response: Do biology, psychology, and culture matter?. Pain Med..

[16] Al-Harthy M., List T., Ohrbach R., Michelotti A. (2018). Cross-cultural differences in types and beliefs about treatment in women with temporomandibular disorder pain. J. Oral Rehabil..

[17] Hardy M.A., Acciai F., Reyes A.M. (2014). How health conditions translate into self-ratings: A comparative study of older adults across Europe. J. Health Soc. Behav..

[18] Achterberg W.P., Gambassi G., Finne-Soveri H., Liperoti R., Noro A., Frijters D.H.M., Cherubini A., Dell’Aquila G., Ribbe M.W. (2010). Pain in European long-term care facilities: Cross-national study in Finland, Italy and the Netherlands. Pain.

[19] Kunz M., Scharmann S., Hemmeter U., Schepelmann K., Lautenbacher S. (2007). The facial expression of pain in patients with dementia. Pain.

[20] Oosterman J.M., Traxler J., Kunz M. (2016). The Influence of Executive Functioning on Facial and Subjective Pain Responses in Older Adults. Behav. Neurol..

[21] Bunk S., Emch M., Koch K., Lautenbacher S., Zuidema S., Kunz M. (2020). Pain Processing in Older Adults and Its Association with Prefrontal Characteristics. Brain Sci..

[22] Bunk S., Zuidema S., Koch K., Lautenbacher S., De Deyn P.P., Kunz M. (2021). Pain processing in older adults with dementia-related cognitive impairment is associated with frontal neurodegeneration. Neurobiol. Aging.

[23] Fleiss J. (1986). The Design and Analysis of Clinical Experiments.

[24] Campbell C.M., Edwards R.R. (2012). Ethnic differences in pain and pain management. Pain Manag..

[25] Dildine T., Atlas L.Y. (2019). The need for diversity in research on facial expressions of pain. Pain.

[26] Ekman P., Blacking J. (1977). Biological and cultural contributions to body and facial movement. The Anthropology of the Body.

[27] Matsumoto D. (1990). Cultural similarities and differences in display rules. Motiv. Emot..

[28] McDuff D., Girard J.M., el Kaliouby R. (2017). Large-Scale Observational Evidence of Cross-Cultural Differences in Facial Behavior. J. Nonverbal Behav..

[29] Lautenbacher S., Kunz M. (2017). Facial Pain Expression in Dementia: A Review of the Experimental and Clinical Evidence. Curr. Alzheimer Res..

[30] Defrin R., Amanzio M., De Tommaso M., Dimova V., Filipovic S., Finn D.P., Kunz M. (2015). Experimental pain processing in individuals with cognitive impairment: Current state of the science. Pain.

[31] Kunz M., Meixner D., Lautenbacher S. (2019). Facial muscle movements encoding pain—A systematic review. Pain.

[32] Kunz M., Mylius V., Schepelmann K., Lautenbacher S. (2008). Impact of age on the facial expression of pain. J. Psychosom. Res..

[33] Defrin R., Beshara H., Benromano T., Hssien K., Pick C.G., Kunz M. (2021). Pain Behavior of People with Intellectual and Developmental Disabilities Coded with the New PAIC-15 and Validation of Its Arabic Translation. Brain Sci..

[34] Lin P.-C., Lin L.-C., Shyu Y.-I.L., Hua M.-S. (2011). Predictors of pain in nursing home residents with dementia: A cross-sectional study. J. Clin. Nurs..

